# Modeling the blood-brain barrier for treatment of central nervous system (CNS) diseases

**DOI:** 10.1177/20417314221095997

**Published:** 2022-05-14

**Authors:** Olivia Rice, Allison Surian, Yupeng Chen

**Affiliations:** Department of Biomedical Engineering, University of Connecticut, Storrs, CT, USA

**Keywords:** Blood-brain-barrier, CNS, drug delivery, brain-on-chip, neurodegenerative disease

## Abstract

The blood-brain barrier (BBB) is the most specialized biological barrier in the body. This configuration of specialized cells protects the brain from invasion of molecules and particles through formation of tight junctions. To learn more about transport to the brain, in vitro modeling of the BBB is continuously advanced. The types of models and cells selected vary with the goal of each individual study, but the same validation methods, quantification of tight junctions, and permeability assays are often used. With Transwells and microfluidic devices, more information regarding formation of the BBB has been observed. Disease models have been developed to examine the effects on BBB integrity. The goal of modeling is not only to understand normal BBB physiology, but also to create treatments for diseases. This review will highlight several recent studies to show the diversity in model selection and the many applications of BBB models in in vitro research.

## Introduction

**Figure 1.**
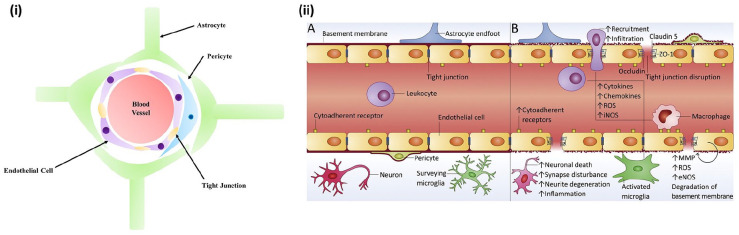
In vivo blood-brain barrier structure: (i) Cross sectional view. (ii) A. View of healthy BBB and B. view of BBB with neurodegenerative disease. Reproduced from Saraiva et al.^2^.

The blood-brain barrier (BBB) is a highly selective, physical layer consisting of mainly endothelial cells that surround the brain, separating the lumen of the cerebral blood vessels and the brain parenchyma.^1,2^ This selectivity is characterized by cellular tight junctions between the endothelial cells that only allows specific neural signaling resulting in blood exchange throughout the central nervous system (CNS).^3^ Although the barrier is composed of endothelial cells, it is surrounded by a layer of pericytes, which are then surrounded by a basement membrane and astrocyte end-feet connections^4^ (Figure 1). Pericytes are vessel wall-associated cells, most often seen in small vessels such as capillaries.^5^ Within capillaries, pericytes exhibit contractile properties, which plays a role in the tightness of the vessels that make up the BBB.^6^ Astrocytes are fundamental in BBB function as they support the transport of ions and water across the barrier. Specifically, the astrocytic end feet express multiple permeable channels that promote this transport.^7^ Kir4.1 channels are one of the channels created at these end feet that play an important role in maintaining potassium levels in the brain, which ultimately impacts the resting membrane potential.^8^ In addition to Kir4.1 channels, the astrocytic end feet form aquaporin-4 (AQP4) channels, which provides selective permeability to water molecules.^9^

Due to the tight junctions that form from interaction of these three cell types, only certain molecules can enter the brain through the BBB. Under normal physiological circumstances, the BBB is permeable to uncharged molecules at a size of 4 nm or less through diffusion of oxygen and nonpolar molecules, as well as lipid soluble molecules like nicotine, alcohol, and caffeine.^10^ Some molecules essential to brain function, such as glucose and amino acids, can pass the BBB through active transport through carrier-mediated transcytosis, receptor-mediated transcytosis, and ion transport.^11^ The low permeability to most substances and high number of tight junctions have created an intricate protective mechanism, but this has negative implications in drug delivery. Nanoparticles have opened the door for treatment options of cerebral diseases, as they have shown great potential in drug delivery to hard-to-reach tissues.^12,13^

Similarly to the restrictions and challenges that face drug delivery techniques to the BBB, modeling the BBB has proven difficult. In vitro models provide a controlled and quantifiable environment for studying the properties of the BBB, but often are not relevant due to their simplicity.^14^ Not only is the model type, whether it be a Transwell model and microfluidic model, important in designing a pertinent BBB model, the cell line selection is also essential in understanding the properties of the BBB. Animal models often do not mimic the qualities needed in understanding the human BBB.^15^ This review will highlight the many types of models and cell lines that have been used to model the BBB, with the goal of presenting the strengths and weaknesses of each model. In addition to typical in vitro modeling, this review will also focus on disease modeling, which also leads into the implications of nanomedicine and drug delivery through the BBB.

### Model validation

**Figure 2.**
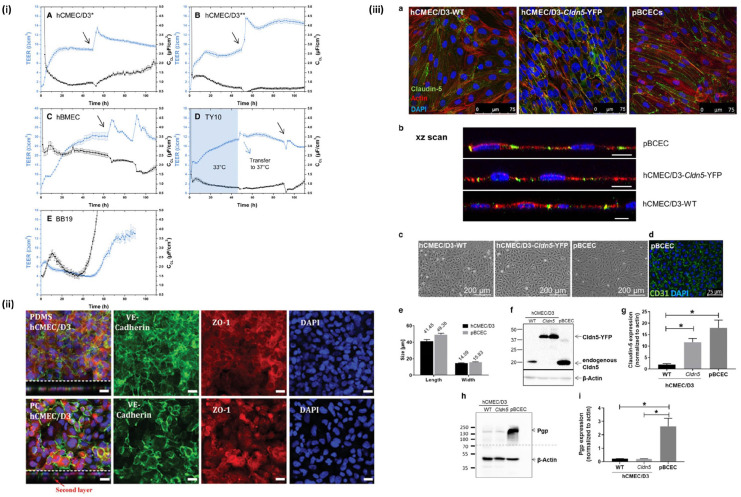
Validation techniques commonly used for BBB models: (i) TEER measurements from four different types of brain endothelial cells. Reproduced from Eigenmann et al.,^51^ (ii) tight junction protein staining of cadherin and ZO-1. Reproduced from Zakharova et al.,^44^ (iii) validation of models using three types of brain endothelial cells with tight junction protein staining and western blot, as well as verification of p-gp expression. Reproduced from Gericke et al.^50^

To model the BBB, it must behave as closely to the native tissue as possible. This includes using methods to confirm the barrier integrity of the model. Transendothelial electrical resistance (TEER) is a quantitative measurement of the tightness of cellular junctions, typically seen in barrier-like structures^16^ (Figure 2.i). The TEER value of an in vitro barrier model is obtained by either measuring ohmic resistance or impedance across a variety of frequencies.^17^ An in vivo human BBB TEER value has not been exclusively explored, but it is believed that mammalian BBBs display TEER values well above 1000 Ω·cm^2^. This value is challenging to achieve in vitro modeling, with it being especially more difficult if immortalized cell lines are used over primary cells.^18^

In addition to determining the TEER value of the BBB, validation of tight junctions can also be explored through permeability studies. These permeability studies require the use of a fluorescent marker, one being sodium fluorescein, which are useful in drug delivery assays.^19^ Other studies have utilized Lucifer yellow and FITC-labeled dextran solutions to validate the limited permeability of the BBB model. Ideally, these solutions should not cross the BBB model, as in vivo, these substances are too large to cross the BBB.^20^

Another method for validating the tight junctions of a BBB model is evaluating tight junction protein expression of claudin-5, zonula occludin (ZO)-1, and occludin. Claudin is a transmembrane protein present at endothelial tight junctions and determines the properties of the barrier of the cell to cell adhesions.^21^ Within the BBB, claudin-5 plays a functional role in the paracellular transport to small molecules.^22^ Due to claudin-5 being a main component in the cell-to-cell adhesions of the BBB, it is a key factor in determining the degree of tightness of the barrier^23^ (Figure 2.ii). ZO-1 is another example of a transmembrane tight junction protein seen in endothelial cells.^24^ In the BBB, ZO-1 acts peripherally within the epithelial cell membrane in order to interact and attach to other membrane proteins, including claudin and occludin.^25^ Similarly, occludin has been seen to increase the TEER value of the BBB.^26,27^

An important validation of the permeability, specifically within drug delivery, is the study of the efflux transporters of the BBB. Transporters within the brain, including glucose transporter 1 (GLUT-1), and organic anion transporting polypeptides (OATPs), which allows molecular transport into the brain, are highly expressed throughout the BBB. Also present at the BBB are ATP-binding cassette (ABC) transport proteins, which are ATP-driven pumps that transport xenobiotics and endogenous metabolites to the brain.^28^ Adversely, P-glycoprotein (P-gp), a well-known ABC protein, actively removes molecules transported into the endothelial cells and is commonly known as a multidrug resistant pump in cancerous tissues^29^ (Figure 2.iii). In modeling the BBB, mimicking these transporters can provide insight to the challenges of drug delivery to the brain.

### Historical timeline of BBB models

**Figure 3.**
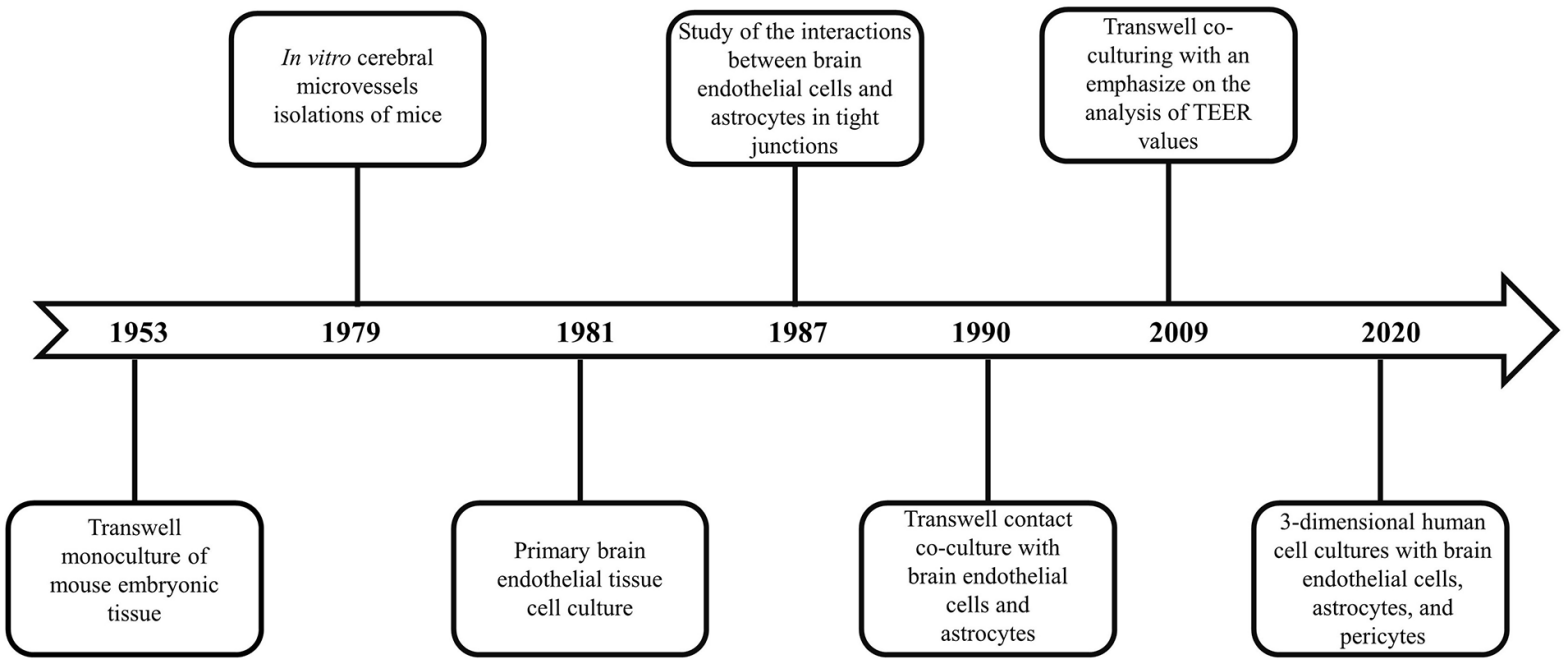
Timeline visualization of the advancements in BBB modeling.^30–37^

Current BBB modeling techniques date back to 1953, when the original monolayer cell culture Transwell system was utilized.^30^ This study cultured embryonic mouse tissues using porous filter membranes to analyze brain endothelial cell permeability.^31^ In the 1970s, scientists began working on in vitro cerebral microvessel isolations of mice in order to study brain endothelial cell cultures.^32^ By the 1980s, the method in extracting brain microvessels was perfected in such a way that primary capillary endothelial cells were able to be isolated and cultured.^33^ In the same decade, a co-culture of bovine brain endothelial cells and rat astrocytes was studied using a coverslip method. This study showed that the interaction between the endothelial cells and astrocytes yielded enhanced tight junctions.^34^ In the 1990s, the co-culture and Transwell model were combined to create a contact co-culture where bovine brain endothelial cells were on the apical side of a Transwell filter and the astrocytes on the basal.^35^ Into the 2000s, models advanced in testing both contact and non-contact Transwell co-cultures with careful analysis regarding the TEER values.^36^ The most recent studies utilize three-dimensional cell cultures, using human brain endothelial cells, astrocytes, and pericytes^37^ (Figure 3). The immortalized cell line used for the human brain endothelial cells is hCMEC/D3 as they exhibit the properties of an in vivo BBB and maintain their structural integrity in a Transwell culture.^38^ In addition to advanced Transwell models, microfluidics have been utilized in order to model the BBB. Microfluidics are seen as a flexible modeling method, as cell culture and mechanical parameters can be altered all within a singular device.^39^ This review will explore a more extensive, in-depth look at each type of model.

## Blood-brain barrier models

Due to the complex nature of the BBB, a perfect model has yet to be created. Oftentimes, studies try to achieve a singular goal in understanding the BBB through in vitro experiments, resulting in models of varying complexity. In addition to model selection and fabrication, the cells used within each model can provide different information. In this section, multiple recent studies will be explored and examined in order to present the numerous BBB model applications. Table 1 provides an overview of both Transwell and microfluidic models discussed in this review.

**Table 1.** Overview of BBB models discussed in this review including design and applications.

References	Model type	Material	Cell type(s)	Application	TEER
Zakharova et al.^44^	Transwell	PDMS membrane	• Human cerebral microvascular endothelial cells (hCMEC/D3)	• Customized membrane inserts for future barrier studies	N/A
			• Human astrocytes	• Microfluidic BBB studies	
Augustine et al.^46^	Transwell	GelMa	• Endothelial cells	• Brain metastasis studies	N/A
			• Astrocytes	• Chemotherapeutic delivery to the brain	
			• MDA-MB-231 breast cancer cell line		
Mendonça et al.^49^	Transwell	N/A	• Human cerebral microvascular endothelial cells (hCMEC/D3)	• Huntington’s disease treatment	20–25 Ω·cm^2^
			• Striatal neuronal cell line (ST14A)	• Nanoparticle synthesis	
Gericke et al.^50^	Transwell	Polyester membrane	• Human cerebral microvascular endothelial cells (hCMEC/D3)	• Use of tight junction proteins in future models	hCMEC/D3: 117 ± 9 Ω·cm^2^
			• Human cerebral microvascular endothelial cells transduced with claudin-5 (hCMEC/D3-Cldn5-YFP)		hCMEC/D3-Cldn5-YFP: 211 ± 8 Ω·cm^2^
			• Primary porcine brain capillary endothelial cells (pBCECs)		pBCEC: 1650 ± 46 Ω·cm^2^
Eigenmann et al.^51, a^	Transwell	PC, PES, or PET	• hCMEC/D3	• Model validation for drug delivery experiments	hCMEC/D3: 5.09–11.9 Ω·cm^2^
			• hBMEC		
			• TY10		hBMEC: 2.79–28.4 Ω·cm^2^
			• BB19		TY10: 4.56–13.0 Ω·cm^2^
Beard et al.^53^	Transwell	N/A	• bEnd.3 cell line	• Validated stem cell model to be reproduced for further studies	N/A
			• Mesenchymal stem cell (MSC)	• Drug delivery through the BBB	
Kuo et al.^54^	Transwell	N/A	• Bovine brain microvascular endothelial cells (BBMVECs)	• Cell culture environment studies	Co-culture: 113.2 Ω·cm^2^
					ACM-treated ECs: 103.3 Ω·cm^2^
			• Human-derived astrocytes		ACM-treated co-culture: 144.4 Ω·cm^2^
Zakharova et al.^63^	Microfluidic	PDMS	• Human cerebral microvascular endothelial cells (hCMEC/D3)	• Reproducible model for future drug delivery studies	N/A
			• Human astrocytes (HAc)		
Lee et al.^65^ and Campisi et al.^66^	Microfluidic	PDMS	• iPSC-ECs	• Nanoparticle transport studies	N/A
			• Brain pericytes	• Patient specific disease modeling and therapeutic development	
			• Astrocytes		
Buzhdygan et al.^67^	Microfluidic	PDMS	• Human cerebral microvascular endothelial cells (hCMEC/D3)	• Further coronavirus BBB studies	N/A
DeOre et al.^69^	Microfluidic	PDMS	• Human cerebral microvascular endothelial cells (hCMEC/D3)	• Further coronavirus BBB studies	TEER was graphically representing to show progressive decrease when exposed to SARS-CoV-2 spike protein
Salman et al.^70^	Microfluidic	PDMS	• Human brain derived microvascular endothelial cells (TY10)	• Imaging of BBB dynamics and transport	N/A
Tu et al.^71^	Microfluidic	PDMS chip with PET membranes	• Human cerebral microvascular endothelial cells (hCMEC/D3)	• Dynamic BBB in vitro modeling	TEER was the main focus of this study, so multiple graphs are provided throughout showing the change in TEER over the course of specified times
Buchroithner et al.^72^	Microfluidic	Acrylic class with PET membranes	• Human vascular endothelial cells	• Imaging of BBB dynamics and transport	N/A
			• Bovine pericytes		
Hudecz et al.^73^	Microfluidic	PDMS chip with custom silicon nitride membrane	• Human endothelial cells	• Customizable BBB modeling	N/A
			• Astrocytes		
Bouhrira et al.^74^	Microfluidic	PDMS	• Human cerebral microvascular endothelial cells (hCMEC/D3)	• Dynamic in vitro BBB modeling	N/A
			• Astrocytes	• Angiogenic disease modeling (i.e. atherosclerosis and aneurysm)	
			• Human coronary arterial smooth muscle cells		
Jeong et al.^76^	Microfluidic	PDMS	• Primary mouse brain microvascular endothelial cell	• Replication microchannel with in vivo like dimensions	N/A
			• Astrocytes		
Kim et al.^77^	Microfluidic	PDMS	• Human bone marrow-derived stem cells (hBM-MSCs)	• Reproducible, optimized cell cultures to be used in further modeling studies	N/A
			• Primary HBMECs		
			• Human pericytes		
			• Human astrocytes		
Santa-Maria et al.^79^	Microfluidic	Polyester	• CD^34+^ cord blood hematopoietic stem cell	• Impacts of fluid flow on BBB integrity	425.5 ± 188.8 Ω·cm^2^
			• Bovine brain pericytes	• Use of stem cells in BBB modeling	
Jeong et al.^81^	Microfluidic	PDMS	• Primary mouse brain microvascular endothelial cells	• Reduction for the need of in vivo animal studies	663–3368 Ω·cm^2^
			• Primary astrocytes		
Yu et al.^82^	Microfluidic	PDMS	• Primary rat neonatal endothelial cells	• Use of primary cells in modeling	TEER values and changes expressed graphically in reference
			• Astrocytes		
			• Pericytes		
Peng et al.^83^	Microfluidic	PDMS	• Human cerebral microvascular endothelial cells (hCMEC/D3)	• Nanoparticle therapeutic transport and treatment	N/A
			• Fetal-hTERT cell line		
			• Immortalized human astrocyte cell line		
Blanchard et al.^89^	Transwell	PES membrane	• Human induced pluripotent stem cells (iPSCs)	• Understanding pathological nature of AD	100 Ω·cm^2^
			• Pericytes		
			• Astrocytes		
Shin et al.^90^	Microfluidic	PDMS	• ReNcell VM human NPCs expressing FAD mutations in the APP gene	• Understanding the biology of AD	N/A
			• Amyloid precursor protein (APP) gene-mutated perivascular neurons	• Drug delivery specific to AD	
Vatine et al.^91^	Microfluidic	PDMS	• Human induced pluripotent stem cells (iPSCs) differentiated into BMEC-like cells (iBMECs)	• Patient specific neurodegenerative disease treatment	1500 Ω·cm^2^
			• Primary human astrocytes		
			• Primary human pericytes		
Pediaditakis et al.^105^	Microfluidic	PDMS	• iPSC-derived brain endothelial cells	• Understanding the neuropathology of Parkinson’s disease	N/A
			• Pericytes		
			• Astrocytes		
			• Microglia		
			• Dopaminergic neurons.		
Cai et al.^108^	Transwell	PET membrane	• Primary rat endothelial cells (rCMECs)	• Understanding the neuropathology of Parkinson’s disease	rCMECs: 392 ± 32 Ω·cm^2^
			• Primary PD rat endothelial cells (PD rCMECs) • Astrocytes	• Treatment of PD	PD rCMECs: 371 ± 29 Ω·cm^2^
Lopalco et al.^110^	Transwell	N/A	• Human cerebral microvascular endothelial cells (hCMEC/D3)	• Nanoparticle transport and treatment of PD	65–89 Ω·cm^2^
Bolognin et al.^112^	Microfluidic	N/A	• Human neuroepithelial stem cell lines (hNESCs)	• Understanding the neuropathology of Parkinson’s disease	N/A
Vakilian et al.^117^	Transwell and Microfluidic	N/A	• Primary human umbilical vein endothelial cells (HUVECs)	• Comparison between two modeling types	TEER values and changes expressed graphically in reference
			• Astrocytes	• Understanding the impact of β-BA on brain metastasis	
Yin et al.^118^	Transwell	N/A	• Brain capillary endothelial cells (BCECs)	• Targeted brain nanomedicine delivery option for cancer patients	N/A
			• Lung cancer cell line H1975		
Seo et al.^119^	Microfluidic	PDMS	• HBMEC cells	• Treatment of glioblastoma	N/A
			• Human brain vascular pericytes (HBVP)	• Transport of chemotherapeutics to the brain	
			• Human astrocytes (HA)		
Kim et al.^123^	Transwell	Polycarbonate membrane	• bEnd.3 cell line exposed to an OGD environment	• Understanding the impact of ischemic stroke on the BBB integrity	TEER values and changes expressed graphically in reference
Al-Ahmad et al.^125^	TCPS and Transwell	N/A	• Human cerebral microvascular endothelial cells (hCMEC/D3)	• Understanding the pathological nature of neuropeptides seen in stroke patients	TEER values and changes expressed graphically in reference
			• Human induced pluripotent stem cell (iPSC)-derived brain microvascular endothelial cells	• Treatment of stroke through the degradation of the studied neuropeptides	

a See paper for comprehensive list of TEER values for each cell type on each type of Transwell insert. The above table only provides a range of the provided values.

b See paper for the comprehensive outline of TEER values and the changes in TEER throughout the time trials.

### Transwell models

**Figure 4.**
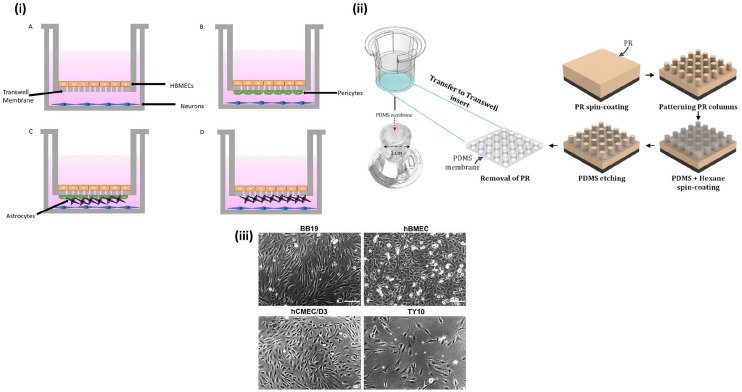
Transwell model for BBB studies: (i) A. Transwell monoculture with human brain endothelial cells (HBMECs). B. Transwell co-culture with HBMECs and pericytes. C. Transwell tri-culture with HBMEC, pericytes, and astrocytes. D. Transwell co-culture with HBMECs and astrocytes, (ii) fabrication of Transwell model with topographical changes for improved BBB integrity. Reproduced from Zakharova et al.,^44^ (iii) four human brain endothelial cell lines cultured for BBB model optimization. Reproduced from Eigenmann et al.^51^

To study the BBB, a porous membrane structure needs to be present to create cell cultures that exhibit the in vivo environment of the barrier, which is often done through a Transwell model.^40^ To model the BBB, multiple Transwell models can be used, with different levels of complexity, the simplest being an endothelial monoculture and higher complexity in co-cultures with multiple cell types.^41–43^ Figure 4.i demonstrates the four basic types of Transwells that are often used. In recent studies, advanced models with co-cultures and biocompatible surfaces have been used and will be further explored in this review.

In the 2021 study by Zakharova et al.^44^ a 2 µm thick polydimethylsiloxane (PDMS) membrane was created at two different pore sizes, 3 and 5 µm. This custom PDMS membrane replaced the typical polycarbonate (PC) membrane, with the main focus of determining whether membrane thickness influenced the integrity of the BBB cell culture. The PDMS membranes fabricated in this study started as a positive photoresist spin coated onto a silicon wafer (Figure 4.ii). The PDMS was patterned with the desired pore sizes using photolithography and then the patterned array is coated with a PDMS and hexane solution. Finally, the new membrane was etched using reactive ion etching and then transferred back into the Transwell insert. After successful transfer of the custom-made inserts, a co-culture of human cerebral microvascular endothelial cells (hCMEC/D3) and human astrocytes was used. One advantage of this porous membrane is that the cell culture can be visualized through phase contrast imaging. This is a non-invasive way to monitor a cell culture without the need for labeling through fluorescence. This technique is not viable with the use of a PC membrane as the light scatters and the state of the cells cannot be determined until after the experiment has been completed.^45^ Prior to studying the permeability of the cell culture, the cell viability was tested using live/dead assays. The main goal of the paper was to create a model with increased cell-to-cell interactions within a culture through the use of controlled pore sizes. Specifically, the cell-to-cell adhesion of hCEMC/D3 cell lines and astrocytes were studied as these are the cells that interact in vitro with the BBB basement membrane. The success of this experiment was determined through the immunostaining of tight junction proteins, including claudin-5, cadherin, and ZO-1 (Figure 2.ii). This study was able to determine that the cell-to-cell adhesions within a BBB model can be increased through the precise control of pore size and reduction of membrane thickness, however, it was also determined that the mechanical properties of the insert material may have influence on the cell culture conditions. It was also mentioned that this insert can be transferred to an organ-on-chip study, as PDMS is often the material selected in the fabrication of microfluidic devices. The potential impact of the custom membrane seen in this study is great, as it can be applied to future BBB Transwell or microfluidic studies, but also any physiological barrier study, including lung models.

While studying the impact of breast cancer of the BBB, Augustine et al.^46^ developed a triple layer Transwell consisting of a gelatin-methacryloyl (GelMa) hydrogel modified Transwell insert, astrocytes, and endothelial cells in order to study the effects of a chemotherapeutic agent against breast cancer cells and the metastasis of the disease cells to the brain. GelMa is a semi-synthetic material often used in hydrogels for drug delivery.^47^ This material is ideal for in vitro studies as the gelatin provides high biocompatibility and the addition of methacryloyl enhances the mechanical property of the material, including increased mechanical strength.^48^ Prior to seeding the endothelial cells and astrocytes, the GelMa solution was coated over the Transwell inserts at two thickness values, 50 and 100 µL. After infiltrating the pores of the Transwell membrane, a photocrosslinker, specifically UV exposure, with the purpose of curing the GelMa layer onto the membrane, was used. The Transwell-GelMa insert was imaged using scanning electron microscopy (SEM) and it was seen that the uncoated inserts had larger, more randomly spaced pores, showing the Transwell-GelMa model has a more controlled pore pattern. After creating the triple layer, non-diseased BBB model seeded with endothelial cells and astrocytes, the study tested the permeability of cancer cells through the fabricated model. To track the metastasis of cancer cells through the BBB, tagged MDA-MB-231 triple-negative breast cancer cells were seeded and tracked through each model. The final aspect of this study consisted of testing the impact of the chemotherapeutic agent cisplatin. It was seen that there was a concentration-decrease in the metastasis of the MDA-MB-231 cancer cells, indicating that if breast cancer is treated early enough with cisplatin, there is a decrease in brain metastasis. As the interest in the treatment of breast cancer metastasis increases, a model such as this one is essential in not only studying the cells, but also the chemotherapeutics. Future studies could explore other therapeutics as this study only focuses on one or they could implement the protocol using a more complex model, for example, creating an in vitro microvessel lined with GelMa through the use of a microfluidic device.

A common issue explored by researchers regarding the BBB focuses on the treatment of the numerous neurodegenerative diseases. Although some of the most common disease states are highlighted in this review, there are many others that have been studied. Huntington’s disease is a genetic neurodegenerative disease caused by a mutation in the huntingtin (HTT) gene, current research explores the use of cyclodextrin nanoparticles (CDs) loaded with siRNA in order to downregulate the mutated HTT gene.^49^ The modified CD-siRNA nanoparticles were synthesized following a previously established protocol and then were characterized using Fourier-Transform infrared spectroscopy (FTIR), nuclear magnetic resonance (NMR), and high-resolution mass spectrometry (HRMS). The BBB in vitro model used was a hCMEC/D3 monoculture on a Transwell insert. The model was validated through TEER analysis and immunohistochemical staining of tight junction protein ZO-1. Once synthesized, the siRNA was FAM-labeled and the transcytosis of the nanoparticles were tracked over the course of 4 h, seeing a gradual increase of the CD-siRNA nanoparticles in the basal compartment of the Transwell. Unmodified CD has been deemed unable to cross the BBB, but when modified, it has been successful. After transport across the monoculture was established, a co-culture with the hCMEC/D3 cells and striatal neuronal cell line ST14A was used to not only study the transport of the nanoparticle across the BBB, but then to simulate to in vivo environment of treating the diseased neuronal cells. Through immunostaining, HTT gene silencing was observed in the co-culture model. This study showed the delivery effectiveness of siRNA through the BBB. It was mentioned that further research is needed in order to optimize the fabrication and delivery of the nanoparticle, but this study is a promising start to understanding the treatment of Huntington’s disease.

The use of Transwells in BBB modeling is not only beneficial in creating a physical barrier through a membrane insert, but they also promote cell-to-cell interaction. Typically, the complexity of the model differs depending upon the study’s goal. Advancements in Transwell modeling allow for more BBB studies to be completed, ultimately enhancing the understanding of the BBB.

#### Cells used in transwell models

An in vivo BBB consists of multiple cell types that all interact to create an intricate barrier. Due to this complexity, selecting the cell types for a BBB model can be challenging. In addition, depending on the Transwell model type, one cell type may only be required. In such cases, the endothelial cells are prioritized. In a recent Transwell model study, hCMEC/D3 cells were compared to a porcine endothelial cell line^50^ (Figure 2.iii). In this particular study, the hCMEC/D3 was transduced with claudin-5. The goal of the study was to show that a human model is suitable in mimicking the BBB. Through TEER measurements, it was proven that claudin-5 had the capability to increase TEER values and lower permeability.

In a 2013 comparative study, four human brain endothelial cell lines were cultured and examined in order to optimize their BBB model.^51^ The main requirement of the comparison was to see which cell line, hCMEC/D3, hBMEC, TY10, or BB19, was best able to form a significant barrier that could be used for drug permeability studies (Figure 4.iii). Multiple different types of cultures were utilized in a Transwell model, with the desired endothelial on the apical side of the porous membrane and for the contact co-cultures, either an astrocyte cell line or pericytes cell line were seeded on the basolateral side of the membrane, and for the non-contact co-cultures, the same astrocyte cell line was seeded on the basolateral side as well. Additionally, a monoculture of hCMEC/D3 was optimized in this study. The main factors considered were the TEER values of each model, and secondarily, the tight junction protein expression was also observed, and it was concluded that the hBMEC were best suited for in vitro BBB modeling.

One major challenge when studying the brain is its very limited allowance of transcytosis.^52^ Due to this, many studies focus on the transcytosis and delivery to the brain. Beard et al.^53^ used bEnd.3 cells, which is a mouse brain endothelial cell line, to co-culture with mesenchymal stem cells (MSCs) in order to determine the transcytosis potential of their fabricated sweet arrow-peptide (SAP) brain delivery vehicle. Using a 3D Transwell model, it was shown that this co-culture produced the highest resistance to molecule diffusion, validated by TEER measurements and permeability assays. Immunostaining of tight junction protein ZO-1 in the bEnd.3 cells was used and confirmed the formation of tight junctions in the model. This co-culture was proven to be significant and reproducible in BBB modeling, which is important for future studies that may want to utilize this Transwell. For this particular study, the means of cell culture and barrier creation were not the main focuses of the paper, but rather supplementary to the delivery vehicle research. The reproducibility of this model is the considerable portion of this study in terms of BBB modeling.

While cell type selection is important in determining the effectiveness of a BBB model, the improvements made to the culture conditions and other additives to the culture environment also have the potential to improve current, established Transwell models. Kuo et al.^54^ showed that an astrocyte co-culture and a monoculture exposed to astrocyte-conditioned media (ACM) had the potential to enhance BBB properties of an often considered simplistic model. The endothelial cell type selected for use were bovine brain microvascular endothelial cells (BBMVECs), which were seeded onto the porous Transwell membrane. For the contact co-culture, human-derived astrocytes were seeded on the basal side of the membrane. In the monoculture, the BBMVECs were cultured in ACM as an alternative to the contact co-culture. When the contact co-culture was combined with ACM, significant increases in TEER were seen, which allows for the conclusion that cell culture condition does impact the properties of the model barrier. Throughout the entire experiment, each model was immunostained for tight junction protein ZO-1, and the corresponding images prove the existence of tight junctions in the model, further validating the TEER values obtained. This information allows for numerous options for future works as cell selection is not only a consideration, but cell culture environment conditions, including conditioned media or exposure to ECM proteins, must also be made

### Microfluidic models

**Figure 5.**
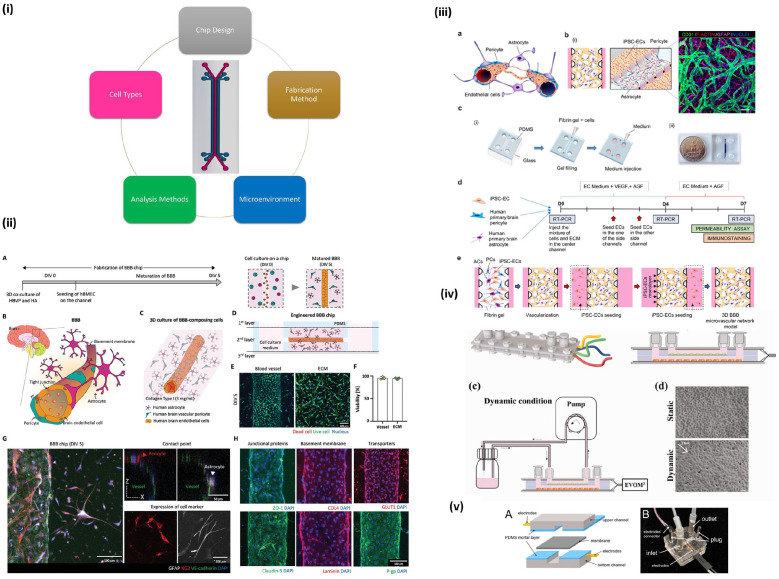
Microfluidic devices used for BBB modeling: (i) example of a two channel microfluidic device and components of tissue models, (ii) 3D culture on chip with various validation methods. Reproduced from Seo et al.,^119^ (iii) microfluidic model using a combination of PDMS, glass, and fibrin gel with a mixture of three cell types. Reproduced from Campisi et al.,^66^ (iv) microfluidic chip using a pump for dynamic flow of media and built-in TEER analysis. Reproduced from Santa-Maria et al.,^79^ (v) model demonstrating ability for continuous TEER measurement. Reproduced from Tu et al.^71^

Microfluidics is a modeling technique that uses and manipulates small fluid channels for a number of applications.^55^ This microscopic modeling technique has many advantages including streamlining advanced biological protocols, reduction in sample size, cost effectiveness, and precise research results.^56,57^ Generally, this technique is referred to as an organ-on-a-chip, and the ultimate goal is to not develop an entire organ model, but rather a simplified version of the main functional unit of the desired organ.^58^ In this way, multiple cell types can be co-cultured, ECM can be simulated and functionalized,^59^ and real-time analysis can be performed. Microfluidic chips are advantageous because there are many tunable parameters, such as biomimetic substrates (for improvement of cellular adhesion),^60,61^ including different microenvironments (to introduce molecules such as growth factors for differentiation and proliferation),^62^ the actual chip design (by changing channel dimensions and flow rate), and the internal analysis methods used (Figure 5.i). When studying the BBB, microfluidics has been popular as they provide multiple options in creating a porous barrier model.

Zakharova et al.^63^ fabricated two, eight-channel microfluidic devices, one with one layer and the other with two layers. The goal of this study was to challenge current BBB models and create a new one that allows for multiple sections of the model to be tested independently of each other. One main issue of using individual systems is that test results are not accurate to in vivo situations. The multiplexed microfluidic device created in this study allowed for higher technical testing, which resulted in better data readouts. To fabricate the microfluidic chip, a PDMS prepolymer was casted with a curing agent and the eight parallel channels were created using lithography processing. This consisted of using elastomeric stamps to print the desired pattern onto a surface.^64^ The device was 3 mm thick, and the inlets and outlets were created using a 1 mm diameter stamp. The two-layer chip had an extra element, as the top and bottom were separated by a porous PDMS membrane. Within the microchannels, hCMEC/D3 and human astrocytes were cultured. After successfully culturing the cells, and by showing that the BBB was mimicked through TEER and permeability assays, the study was able to prove that this modeling technique was not only successful in creating ideal barrier conditions, but is also more reproducible than other current models.

When exploring drug delivery to the brain, failure has often been seen due to the lack of complex brain models. In order to study the transport of polymer nanoparticles, Lee et al.^65^ used a previously fabricated 3D microfluidic model (Figure 7.iii). The microfluidic device was composed of a PDMS scaffold with a single microchannel and two fluid channels. The device was then cultured with human endothelial induced pluripotent stem cells (iPSC-ECs) with pericytes and astrocytes. In another study that utilizes the same modeling technique, it was concluded that the 3D microfluidic model presented a more realistic representation of the transport of nanoparticles through fluorescence and permeability studies^66^ (Figure 5.iii). The use of iPSC cells demonstrated an additional research factor, as they allow for patient specific modeling, with possibilities centering around patient specific nanoparticle therapeutics. The specific microfluidic model used in these two studies has the potential to be further validated and then used in patient specific disease modeling and treatment studies. This combination of stem cell models with drug delivery research is the future of BBB modeling and has the potential to result in specialized nanomedicine treatments.

As the rise of coronavirus impacts individuals worldwide, there has been a huge increase in research focusing on the effects of the virus on the body. A new study focused on how the coronavirus alters the BBB through the use of in vitro 3D modeling.^67^ Using a previous fabrication method, the microfluidic device was created using a silicon mold and a combination of unpolymerized photoresist and PDMS.^68^ The device microchannels were then injected with a collagen-based hydrogel. The hCMEC/D3 cell line was seeded and cultured in one channel of the microfluidic device and after proper environmental conditions were created, the cells were exposed to the viral protein SARS-CoV-2 subunit S1. Immunofluorescence was also conducted in staining of ZO-1 to localize the tight junctions within the microvessel model. To monitor the effects of the virus on the tight junctions of the BBB, permeability testing was conducted, specifically real-time TEER measurements and analysis. They were able to conclude that when exposed to the viral protein, there was destabilization of the BBB and inflammation of the endothelial cells. The work done in this study could be further explored, as a co-culture or tri-culture model exposed to the SARS-CoV-2 subunit S1 should be used. This would provide a more accurate BBB disease model and with the public health concern of the long-term effects of the coronavirus, could provide better understanding of the virus’s effect in the CNS.

To further explore the use of microfluidic models in COVID-19 research, a 3D in vitro model was utilized to observe the impact of the SARS-CoV-2 spike protein of BBB dysfunction, specifically through RhoA activation.^69^ The microfluidic devices were created following a previous protocol and the channels were seeded with a hydrogel containing hCMEC/D3 cells. Prior to exposing the channels to SARS-CoV-2 subunit S1, model validation using permeability assays and TEER measurement. The exposure was then conducted in conjunction with angiotensin-converting enzyme 2 (ACE2), which is found throughout the endothelium of the body and mediates spike protein binding. To determine the impact of the SARS-CoV-2 S1 spike protein, it was observed that only within a dynamic, fluid model, was there an increase in ACE2 in the presence of the spike protein. Then, to study the function of ACE2 in the BBB model, they performed an ACE2 knockout experiment. It is suggested, through the use of immunofluorescence of tight junction protein ZO-1, that ACE2 does impact the integrity of the tight junctions of the BBB. RhoA has been previously observed to regulate endothelial tight junction integrity and disrupt the vascular nature of endothelial barriers, including the BBB. To determine whether spike protein binding to ACE2 initiates activation of RhoA, ELISA analysis was used to measure the active form of RhoA within the BBB model. The results proved their hypothesis that the spike protein activates RhoA, which subsequently indicates decrease in barrier strength and integrity. The results of this study are paramount in understanding the SARS-CoV-2 S1 spike protein and what it impacts within the brain. The use of a dynamic model is promising, but the use of a monoculture does not provide a complete picture of the cell types present within the BBB. In addition, only one subunit of the spike protein is tested, and if a complete understanding of the spike protein is desired, multiple experiments using a more complex model with all spike subunits should be conducted.

In a study from Salman et al.,^70^ an open microfluidic device was explored in modeling the BBB and compared to the commonly used closed models, previously examined in this review. The overall goal of the research was to overcome the current limitations of other BBB models using an open system that allowed for constant and direct access to the reagents embedded in the device. The microfluidic chip was fabricated with one hollow channel with the intention of cell seeding. Similarly to previous studies, the physical chip was fabricated using PDMS by means of photolithography. The cells selected were human brain derived microvascular endothelial cells (TY10). One of the main goals of study was to show that the open model allows for high resolution imaging, resulting in the use of spinning disk confocal imaging, lattice light sheet microscopy (LLSM), and transmission electron microscopy (TEM). With these imaging techniques, the researchers were able to conclude that the use of the open model not only enhanced the imaging required when studying the BBB, but it also allowed for better overall control of the vascular functions of the microfluidic device.

As previously discussed, measuring and achieving high TEER levels is essential in creating an effective BBB model. In a study conducted by Tu et al.,^71^ an organ-on-chip design was utilized with TEER electrodes built into the design. The chip structure was fabricated out of PDMS using soft lithography and microelectromechanical processes. The chip consisted of a top and bottom layer with an area for the electrodes in the middle. The two sections were brought together using an PDMS-toluene adhesive (Figure 5.v). During the fabrication process, the flow and shear stress of the channels were managed, as these factors can affect the cell survival of the endothelial cells used in this design. The goal of this study was to conduct real time TEER measurements using the electrodes embedded in the chip. This was proven successful and allows possibilities for conducting fluid, real-time measurements, and experiments.

To understand transport of molecules and particles to the brain, thus permeating the BBB, imaging is necessary. To achieve this, in vitro modeling is required to mimic the BBB and then analyze the transport across the BBB.^72^ One study created a two-channel microfluidic device, with human vascular endothelial cells cultured in one channel and pericytes. The outer layer of the chip was composed of acrylic glass, which increased the mechanical soundness of the chip. The two channels were separated by a PET-foil which promoted cell survival. Because the chip was designed to image the interactions of the in vitro BBB, observation windows were created through the PET-foil. Often, optical distortion is seen when a PET layer is incorporated into a BBB microfluidic design. This study created custom holes in the PET-foil, the observation windows, using a laser cutting technique. The observation windows yielded high resolution images of the diffusion of particles. In addition to successful imaging, the study also conducted diffusion analysis and single molecule tracking across the in vitro BBB.

In modeling, a major goal is reproducibility of the design with meaningful validation results. As seen in some studies, microfluidic chip designs and fabrications are taken from a previously established model. In previous studies, a PET membrane has been often seen in both Transwell and microfluidic models, but a 2020 study utilized a custom, layered chip design to explore the use of a silicon nitride membrane.^73^ The top half of the chip, which held the cell cultures and media, was made from a PDMS block and the silicon nitride was incorporated into membrane chip layers. The study utilized a co-culture of human endothelial cells and astrocytes, and the silicon nitride membrane was coated with fibronectin and collagen to promote adhesion to the membrane. The silicon nitride membrane allowed for high quality imaging during transcytosis experiments, which also allowed for nanoparticle transport analysis.

When progressing in BBB modeling, there are numerous considerations that must be made. As previously discussed, culture conditions and TEER measurements are the most novel and most studied aspects of a BBB model. As researchers continue to understand the BBB, they begin to advance from these well-established techniques. The discussion of creating a 3D, dynamic model better mimics the BBB, as they create a microvessel environment that includes the flow of fluid. In the case of the BBB, it is surrounded by cerebral fluids and blood, and in Bouhrira et al.,^74^ the fabrication and characterization of a 3D microfluidic model that provides physiologically relevant flow rate waveforms were explored. The microfluidic devices used throughout the study were fabricated using a previously established protocol, which details the soft lithography of PDMS.^68^ The PDMS was used to cast both positive and negative molds of the cerebral bifurcation geometry that was desired for this experiment. The hydrogel reservoir was washed with sulfuric acid to promote gel adhesion and then injected with a collagen type 1 hydrogel solution. Within the hydrogel solution, a coculture of astrocytes, human coronary arterial smooth muscle cells, and hCMEC/D3 cells were seeded to create the cellular vessel microenvironment. To create the in vitro flow system, a peristaltic pump system control using Arduino based software and a DC voltage motor was implemented.^75^ To begin the measurements of the time-dependent flow waveforms, a programmable linear actuator was implemented into the flow system and was characterized using computational fluid dynamic (CFD) velocity contour plots. A number of physiological waveforms were created using time (in seconds) as the independent variable and flow rate (in mL/min) as the dependent variable. To determine the physiological flow’s impact on barrier integrity, tight junction protein staining, specifically ZO-1 immunofluorescence, and permeability tests were conducted. It was concluded that barrier integrity and function was reduced when exposed to physiological flow. This study was the first of its kind, as it was the first demonstration of a 3D in vitro model with separated, physiological flow. Although the results convey that cerebral blood flow promotes BBB breakdown, further studies specifically looking at cerebral blood flow pathologies such as atherosclerosis and aneurysm must be conducted.

As previously discussed, validating an in vitro BBB model is essential in determining whether the model can provide valuable, in vivo results. Although TEER values and permeability assays are the most used, an in vivo BBB endures many other environmental conditions, including cerebral fluid flow and internal stresses. Jeong et al.^76^ set the goal of creating a numerical approach-based simulation in order to quantify the shear stresses of the brain utilizing a microfluidic chip. The PDMS microfluidic model consisted of an upper and lower half, with four microchannels in each half. The upper channel represented the luminal channel, with primary mouse brain microvascular endothelial cells seeded within the channel. The bottom channel represented the abluminal channel, with astrocytes seeded within the channel. A polycarbonate membrane separated the upper and lower channels and the porous nature of the membrane allowed for fluid flow between the channels. When analyzing the shear stresses within the channels, a number of variables were considered, including, the porosity of the polycarbonate membrane, the viscosity of the fluid, the volume flow rate, and the geometry of the channels. The channel length remained constant throughout all experiments because the channel length had no impact on the shear stress. The first experiment consisted of determining how the boundary width of the microchannels impacts the shear stress. The test was conducted with four different membrane porosity values, and it was determined the microchannel boundary width should not exceed 1.6 mm, showing that there was a decrease in shear stress as the channel width increases. Similarly, microchannel height was examined and similar results were seen, as the height should not exceed 0.8 mm or else shear stress will decrease greatly. The porosity also presented details regarding shear stress, as lower membrane porosity values yielded higher shear stress values. The numerical approach-based simulation in this study optimized the creation of a microfluidic device that provides in vivo shear stress results. The quantified values of the channel width, height, and porosity can be used in any future BBB in vitro models, as the dimensions are proven to provide a meaningful microchannel.

#### Cells used in microfluidic models

Similar to the selection of cells in Transwell models, the selection of cells to culture can be equally challenging. Due to the three-dimensional nature of a microfluidic model, multiple cell types can be cultured within the fluid channels to mimic the BBB. In a study from Kim et al.,^77^ the use of human bone marrow-derived stem cells (hBM-MSCs) in addition to human brain endothelial cells, astrocytes, and pericytes created a stronger, more accurate BBB model. Using a PDMS microfluidic device, five different cell cultures were compared: primary HBMECs, HBMECs and human pericytes, HBMECs and hBM-MSCs, HBMECs, human astrocytes and human pericytes, and HBMECs, human astrocytes, and hBM-MSCs. All cell cultures were suspended in a fibronectin hydrogel. The goal of the study was to demonstrate that hBM-MSCs have better perivascular vessel construction capacity than human pericytes during in vitro modeling. Through tight junction characterization, it was concluded that the hBM-MSCs were better for modeling BBB pericytes than human cell lines, as they had a higher expression of angiogenesis proteins, including vascular endothelial growth factor (VEGF). This information is essential for BBB modeling, as the promotion of angiogenesis allows for a more accurate BBB model.^78^

In addition to hBM-MSCs, other stem cells have been cultured and differentiated in order to model the BBB using a microfluidic chip. Santa-Maria et al.^79^ utilized CD^34+^ cord blood hematopoietic stem cells in their 2021 study. This method of culture was adapted from a 2014 study as this type of cell extraction requires collection of umbilical cord blood and informed consent for the infant donor’s parents.^80^ The stem cells were then differentiated to human endothelial cells (hECs) through the exposure of VEGF for 15–20 days. Once differentiated, the hECs were seeded in the polyester microfluidic chip and co-cultured with bovine brain pericytes. The chip had an upper and a lower compartment, where the hECs were cultured in the apical and pericytes in the basal. The specific goal of the paper was to study how fluid flow generally affects brain endothelium barrier properties, which was why a less specific endothelial cell selection was effective. Through the analysis of BBB related genes, the study was able to conclude that fluid flow increased barrier properties, indicating that fluid flow is essential when modeling the BBB (Figure 5.iv).

Although human derived cell cultures have proven to be effective in modeling the BBB, animal cultures have also closely mimicked the BBB. In a 2018 study from Jeong et al.^81^ primary mouse brain microvascular endothelial cells were co-cultured with primary astrocytes. The microfluidic device was fabricated with two microchannels that came together to form one large BBB unit. In addition to the culture, an extracellular matrix was also simulated using fibronectin or matrigel. To confirm barrier functions, immunofluorescent staining, TEER measurement, and permeability assays were used. The major goal of the paper was to show that an in vitro model can help minimize the need for in vivo animal studies. The paper hopes that the techniques used for primary animal cells can be transferred for use with primary human cells, which will open a variety of options for patient specific treatments.

Another animal microfluidic model utilized primary rat neonatal endothelial cells, astrocytes, and pericytes.^82^ The study detailed the acquisition methods of the primary cells, which requires more steps than cell lines. After euthanization, the rat brains were removed and treated with collagenase to separate the cell types of the brain. After separation, the cells were filtered using a nylon filter. The microchannel of the chip was coated in collagen, and the endothelial cells and pericytes were seeded onto the surface to the channel. The collagen gel that lined the channel had the astrocytes embedded within it. It was seen that the primary cells mimicked in vivo environments better than immortalized cell lines because the phenotypic qualities of the endothelial cells were not lost as they normally are in cell lines. Primary cells also better promote vessel formation, which creates a better BBB model. Although primary cells have a multitude of advantages, the time-consuming nature of obtaining the cells may not be necessary for some studies, which is why they are not always used.

In contrast to some of the above studies which prove that animal models can be effective in a microfluidic model, in certain applications, they are not ideal. Peng et al.^83^ explored drug delivery to the CNS using nanoparticles. Due to issues regarding animal cell cultures in drug delivery including ethical issues and differences in the cellular makeup between species, this study uses all human-derived cell types. For the endothelial cell culture, the hDMEC/D3 cell line was used. The chip was composed of three channels, and the endothelial cells were injected into the blood channel of the chip. In addition to endothelial cells, the fetal-hTERT cell line, an immortalized human astrocyte cell line, were cultured and then injected into the blood channel. The third cell culture was a combination of human immortalized pericytes and a human glioblastoma cell line. The glioblastoma cell line was essential in this study as the goal was to explore the treatment of brain diseases through the use of nanoparticles. The channels were also coated in ECM proteins to promote cell adhesion within the channels. Using in situ modifications and verification through permeability tests, the researchers were able to mimic in vivo BBB environments and show nanoparticle transport within the model.

## Disease modeling

One major advantage when using in vitro models is that the environment and culture conditions are closely controlled. This is beneficial when creating an abnormal cellular environment. Brain diseases are often considered complex and suffer from a lack of research. There have been numerous studies that have tried to understand how different diseases affect the brain. Specifically in this review, different disease states will be investigated and their impact on the BBB will be highlighted. In addition, the model and cell culture conditions will be explored, further describing what was altered from the typical BBB model to make it a disease model.

### Alzheimer’s disease and dementia

Alzheimer’s Disease (AD) is the leading cause of dementia affecting over 6.2 million Americans and millions more worldwide.^84^ To date, there is no effective treatment for AD to subside symptoms nor to prevent disease progression. As life expectancy continues to increase, AD patient numbers will increase exponentially to 152 million worldwide by 2050.^85^ The characteristic pathology of AD includes the extracellular accumulation and aggregation of β-amyloid peptide (Aβ), neurofibrillary tangles (NFTs), gliosis, and neuronal loss due to neuroinflammation.^86^ It is unclear whether the lack of Aβ clearance causes BBB dysfunction, or whether BBB dysfunction hinders Aβ clearance. Mostly, there are no identifiable pre-markers of AD, however polymorphism of the apolipoprotein E (ApoE) gene is a hallmark genetic risk for AD due to evidence of early onset Aβ accumulation and NFTs.^87^ Many BBB models for AD focus on Aβ-induced pathology to study aggregation and clearance mechanisms.^88^

**Figure 6.**
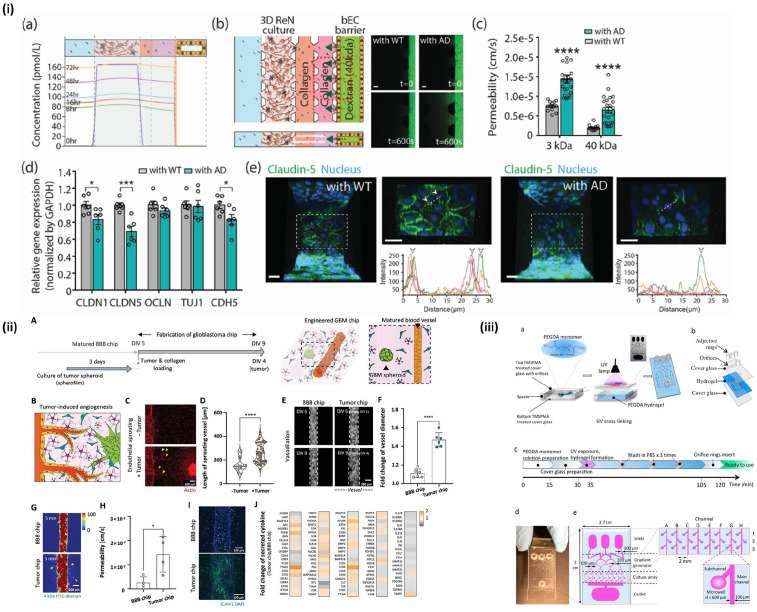
Studies utilizing disease models of BBB: (i) comparison of healthy (WT) BBB with Alzheimer’s disease (AD) BBB in permeability and expression of tight junction proteins. Reproduced from Shin et al.,^90^ (ii, iii) BBB models utilizing tumor spheroids within the tissue chip. Reproduced from Seo et al.^119^ and Fan et al.^140^

The use of induced pluripotent stem cell (iPSC)-derived BBB cells from AD patients has shown promise as the AD pathology is translated into monolayer culture, especially from patients with different ApoE isoform genotypes. By using iPSC-derived BBB cells from patients with ApoE polymorphisms, Aβ aggregation was amplified.^89^ In this study, the ApoE4/4 genotype showed the most dramatic Aβ aggregation when iPSC-derived BBB cells were cultured in Aβ conditioned medium. Most recently, microfluidic devices have been used to study AD, specifically with human BBB-on-a-chip models to identify AD biomarkers and pathogenic mechanisms by introducing Aβ directly, or by introducing a mutation to AD neurons to overproduce Aβ. Shin et al.^90^ created a chip with a luminal endothelial monolayer of immortalized hBMECs and 3D culture of amyloid precursor protein (APP) gene-mutated perivascular neurons to allow for enhanced aggregation of Aβ (Figure 6.i). Through permeability studies and quantification of tight junction proteins, it was found that APP gene mutation resulted in increased BBB permeability. When treated with inflammatory cytokines, such as tumor necrosis factor α (TNF-α), interleukin-1 beta (IL-1β), and IL-8, through the vascular or parenchymal channel, neuroinflammation was seen to increase BBB permeability by altering expression of ZO-1 and increasing dextran permeability.^91^ This also showed neuroinflammation on the brain side of the chip, demonstrating the effectiveness of this model to resemble AD pathology.

**Figure 7.**
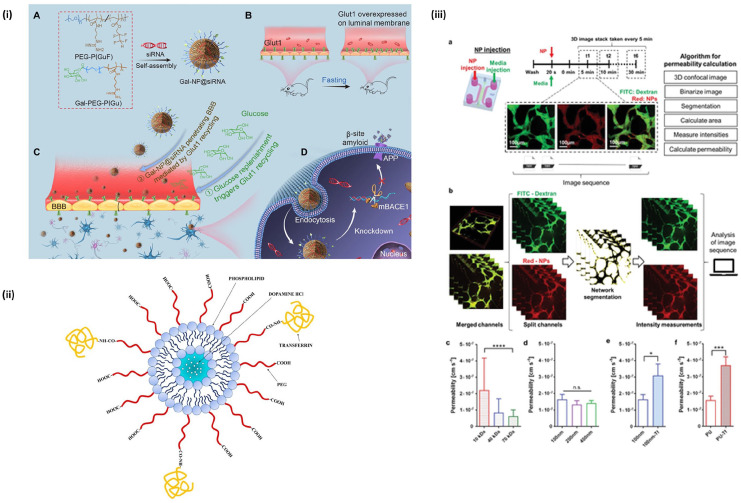
Nanoparticles for BBB drug delivery: (i) example of a NP used for RNAi therapy in AD treatment. Reproduced from Zhou et al.,^134^ (ii) a lipid nanoparticle with transferrin ligand for brain endothelial cell targeting. Reproduced from Lopalco et al.,^110^ (iii) study using machine learning for optimization of NP delivery. Reproduced from Lee et al.^65^

Drug screening and treatment research for AD is also made possible by the aforementioned BBB models. Specifically, RNA interference (RNAi) therapy has been studied due to its potential for disease progression intervention and low effective dose.^92^ However, RNAs are prone to degradation in the body and require vehicles for effective delivery,^93^ especially in tissues with low vascularity or permeability.^94^ Previously, viral vectors were most commonly studied as delivery vehicles for RNAi in AD models, but more recently, nanoparticles have been explored for their targeting specificity, longer circulation lifetime, decreased immunogenicity, and tunability for specific applications, such as crossing the BBB.^95^ β-site APP cleavage enzyme 1 (BACE-1) is a common therapeutic target for AD as it is the enzyme responsible for cleaving APP into Aβ. In a recent study, a glycosylated nanodelivery system was developed which used the glucose transporter-1 (Glut1) receptor for facilitated BBB penetration. The nanodelivery system contained a “triple-interaction” stabilization method that demonstrated high stability in blood circulation and high brain accumulation compared to its cationic polymer counterparts. This nanovehicle was loaded with BACE-1 siRNA and showed not only a decrease in BACE-1 mRNA, but more importantly, a significant decrease in Aβ plaques derived from APP cleavage in AD mice^96^ (Figure 7.i). In another study, magnetite nanoparticles coupled with OPSS, PEG, and NHS were loaded with BACE-1 siRNA and delivered to HFF-1 cells. Cells treated with the magnetite nanoparticles showed significant decrease in BACE-1 expression compared to control cells, showing effective siRNA delivery and potential for BBB penetration.^97^ While knockdown of genes responsible for Aβ accumulation is successful in vivo, it may also be useful to focus on genes for neuron regeneration, such as brain derived neurotrophic factor (BDNF) and nerve growth factor (NGF).^98,99^ In this way, therapeutics can be focused on both prevention and reversal of disease progression.

### Parkinson’s disease

After AD, Parkinson’s disease (PD) is the second most common neurodegenerative disease, often occurring as individuals age.^100^ Technically, PD is the loss of neurons in the substantia nigra compacta, which damages the nigrostriatal pathway in the brain.^101^ Neuron loss is the major pathophysiology outcome of PD, but neuroinflammation and tremor are seen as well.^102^ There have been studies that show BBB damage when PD is present, but there is limited understanding of what the exact root cause is.^103,104^ While there is a lack of understanding on how the disease impacts the BBB, there are also challenges in determining effective drug delivery techniques for the disease.

In an attempt to understand the damaging factor of PD specifically focusing on the BBB, Pediaditakis et al.^105^ investigated alpha-synuclein (αSyn), a protein that accumulates in patients with PD, causing disease-state pathology. This specific protein is believed to cause the build up of Lewy bodies, a protein complex seen in PD that is one of the main markers of disease and cause of memory loss and lack of movement control.^106,107^ In order to study the effects of the αSyn protein, the researchers created a microfluidic chip with human iPSC-derived brain endothelial cells, pericytes, astrocytes, microglia, and dopaminergic neurons. The dopaminergic neurons are commonly found in the substantia nigra compacta, which means PD disease has an immense impact on them in vivo. The chip was fabricated using PDMS and broken up into two sections, a brain channel and vascular channel, which were separated using an ECM-type model made up of collagen, fibronectin, and laminin. The brain channel was composed of neurons and microglia while the vascular channel was composed of endothelial cells. The model was validated prior to exposure to the αSyn using tight junction proteins such as claudin and occludin. Once exposed to the αSyn fibrils, tight junction derangement and compromised BBB permeability was observed. In addition to having a successful PD model, the study also presented the αSyn levels could be controlled through the possible therapeutic use of autophagy inducer trehalose. To properly treat a neurodegenerative disease, it is important to have an understanding of each disease-causing agent and how it may impact the diseased organ. They were able to quantify αSyn’s influence, providing a model that can be replicated for further investigation of PD.

Using a Transwell model, Cai et al.^108^ primarily focused on the comparison of normal rat endothelial cells to the PD rat endothelial cells and the drug delivery potential of each model in the treatment of PD. In order to create the PD rat model, it was injected with 6-OHDA, a neuron toxin that attacks the same areas of the brain as PD.^109^ Four total Transwells were made, two contact cultures, one that was the PD primary rat brain endothelial cells, astrocytes, and primary rat endothelial cells, and two non-contact cultures with PD primary rat brain endothelial cells, astrocytes and primary rat endothelial cells. In order to analyze the effect of PD, TEER was measured and ABC transporter assays were conducted. The results of the study showed that the non-contact PD model had the lowest TEER values and highest permeability, but the contact PD model had comparable TEER values and permeability to the normal models. This result could be caused because of the model selection or the disease-state, so in order to make concrete conclusions regarding the cell cultures, further research is needed.

In the 2018 study, Lopalco et al.^110^ examined the drug delivery possibilities of dopamine through the BBB. PD attacks the dopamine centers of the brain, resulting in neuron loss, so this study looked to deliver this neurotransmitter through liposomes (Figure 7.ii). Liposomes are often used in nanomedicine as they have a similar structure to the lipid bilayer of cells and can hold a variety of substances in an aqueous solution.^111^ Using a hCMEC/D3 cell monolayer, permeability and transport experiments were conducted. In a Transwell system, the liposome nanocarriers were placed on the apical side of the monoculture and successful transport was determined if the nanocarriers were detected on the basolateral side of the Transwell. The main goal of the study was to determine if transferrin functionalized liposomes were more effective in transport across the BBB than a non-functionalized carrier. The permeability of the functionalized liposome was much higher than the non-functionalized, but due to the model being a non-diseased monoculture, conclusions as to whether this transportation technique would be effective in a PD patient cannot be made. Since this study had more of a focus on the fabrication of the nanocarrier, further research could be done in more complex BBB models, with the possibility of disease-state exposure in order to make more accurate conclusions.

As described previously, the use of a 3D microfluidic model creates a more advanced representation of the BBB. In the case of PD, this is very beneficial in the understanding of specific drug development for this complex disease. In Bolognin et al.,^112^ an optimized PD microfluidic model was created and then validated through imaging. The cells used in this experiment were differentiated iPSC cells, specifically from the human neuroepithelial stem cell lines (hNESCs), that were exposed to the LRRK2-G2019S mutation, a disease-causing agent in PD. Culturing of the iPSC cells and creation of the microfluidic device were both derived from previous studies. Once the cells were properly differentiated and seeded within the microfluidic model, time studies were completed to determine when cell death occurs after being exposed to the PD mutation. During these time trials, mitochondrial function of the cells was also observed, as research shows mitochondrial dysfunction in relation to PD. It was observed that the neural mitochondria had a progressive reduction in number and morphological changes were observed. After the time-dependent trials were completed, a proof-of-concept drug screening across the BBB model was conducted. It was seen that when the disease model was treated with LRRK2 kinase inhibitor Inh2, some phenotypic recovery was observed. The final test seen in this study may be the most impactful, as patient specific models created with donor cells were created and tested. It was determined that the genetic background of the cells has an effect on the PD disease outcomes, indicating that PD is patient specific. This study has an interesting outlook, as they deem their experiments as successful, but also recognize that much more research needs to be done. Co-cultures must be explored in order to create a more advanced model. In addition, further patient specific drug delivery testing must be completed, as it is seen that LRRK2 kinase inhibitor Inh2 treatment works for cell lines but may not work with primary PD cells. This study is a meaningful start for the development of gene treatment for PD, but further testing and model validation must be completed.

### Cancer

Cancer remains to be one of the largest issues facing individuals as it is the second leading cause of death in the United States.^113^ Specifically regarding brain tumors, there is limited understanding in detection, and treatment options are often invasive, including surgical removal and radiation treatment.^114^ To develop brain cancer therapies, researchers are developing new nanotechnologies to deliver treatments to the brain, including gene therapies, anti-angiogenic therapies, and thermotherapies.^115^ To successfully deliver a cancer drug to the brain, a proper cancer model must be made and studied. Due to the BBB being the main protective barrier, it is essential to understand how cancer possibly impacts the permeability.^116^

In a recent microfluidic and Transwell study, a BBB model was used to see the effects of β-boswellic acid (β-BA) on reducing the metastasis of breast cancer to the brain.^117^ For both the microfluidic and Transwell models, primary HUVECs and astrocytes were co-cultured to create an in vitro barrier. In addition to β-BA, cisplatin, another chemotherapy drug, was used in cytotoxicity, protein, and migration assays. After each model was exposed to cancer cells and the anti-cancer agents, the results of the static Transwell models and dynamic microfluidic models were compared. It was seen that β-BA was not only successful in killing the cancer cells, but exposure also increased the barrier integrity. The overall experiment was successful, but it was noted that due to the preliminary nature of this study, further research must be done to prove the effectiveness of β-BA.

Many studies, like the above mentioned, focus on the spread of other cancers to the brain. Yin et al.^118^ investigated liposome nanocarriers and their BBB permeability for their treatment in the metastasis of small lung cancer cells. This study was unique to others in this review as it explored both in vitro and in vivo experiments. Liposomes were developed to penetrate the BBB as they were coated in transferrin receptor (TfR)-binding T12, which allowed for higher permeability of the liposome. To create a diseased environment, a Transwell co-culture with brain capillary endothelial cells (BCECs) and a lung cancer cell line, H1975, were used. The study’s focus was to introduce a new method of non-chemo cancer treatment for patients with epidermal growth factor receptor (EGFR) tyrosine kinase inhibitors (TKI) mutations. The cancer cell line was mutated using EGFR^T790M^ and the in vitro results were promising. The liposomes were not only able to cross the BCEC layer, but then able to treat the EGFR^T790M^ mutated H1975 layer. Due to this being one of the first studies of its type, these results are promising in the development of a targeted brain nanomedicine delivery option for cancer patients.

The largest barrier in the treatment of any disease of the CNS, brain tumors included, is the lack of BBB penetration of pharmaceutical drugs. To predict the cellular response to pharmaceuticals, a 3D in vitro glioblastoma model was created.^119^ This BBB chip utilized three layers, the top layer acted as a lid and the bottom included a sliding glass. The middle layer was fabricated from PDMS and included three parallel microchannels where a mixture of human brain vascular pericytes (HBVP), human astrocytes (HA), and collagen were injected into the microchannels (Figure 5.ii). After the collagen had gelated into the microchannels, HBMEC cell suspensions were added into the microchannels. Immediately, the HBMEC cells adhered to the channel due to the collagen coating the channel. Prior to introducing tumor conditions to the model, a number of validations were done, including tight junction protein validations and BBB transporter validations. The chip exhibited BBB characteristics including expression of proteins including ZO-1, claudin-5, and VE-cadherin, and transporters, namely GLUT1 and P-glycoprotein. After typical barrier permeability characterization was completed, the functional model was introduced to glioblastoma (GBM) spheroids in order to determine how a tumor microenvironment (TME) impacts the BBB and drug delivery to the brain (Figure 6.ii). Two types of GBM cell lines, T98G (TMZ-resistant cells) and U87MG (TMZ-sensitive cells), were cultured as spheroids. Each spheroid was labeled and then injected into the hollow channel. The first change that was observed within the model was the formation, damage, and dysfunction of new angiogenic vessels within the model after exposure to the GBM tumors. The pretumorous model exhibited little to no vessel formation, but when exposed to the TME, more vessels not only formed, but the vessels appeared to be more dilated than expected. The tumor spheroids themselves also exhibited morphological changes, when exposed to the BBB microenvironment, an increase in tumor growth and invasion was observed. The last assessment of this 3D model was to investigate the impact of chemotherapies when introduced to the TME within the microvessel. It was observed that the barrier inhibits drug delivery, which allowed tumors to become more resistant to the drug. The use of barrier opening agents such as mannitol and gintonin were used in a drug delivery experiment to observe rapid delivery of chemotherapies through the barrier. This study served as not only a means of BBB modeling, but opened possibilities in disease modeling and treatment. The findings in this study can be utilized in the treatment of other CNS diseases, as the coupling of barrier-opening agents and therapeutics should be further observed.

### Stroke

Strokes are the fifth leading cause of death in the United States and have multiple risk factors. The damage to the BBB caused by strokes can cause increased risk for hemorrhage in the future.^120^ The disruption of the BBB is the biggest pathophysiology outcome of a stroke and the change in the cerebrovascular has the ability to cause post-stroke pathology.^121^ The BBB permeability is initially increased, but after a period of time the baseline permeability is often achieved.^122^ The following studies attempt to mimic a stroke BBB in order to further understand its effect on the BBB.

Kim et al.^123^ studied the specific outcome of autophagy in a OGD stroke environment BBB model. The process of autophagy consists of self-consumption at the cellular level and is often present for targeted molecules to be degraded for the purpose of energy.^124^ In the study, the primary goal was to determine whether autophagy was present at the BBB after a stroke was experienced. To do this, bEnd.3 cells were cultured and exposed to an OGD environment. The monoculture was created using a Transwell model and went through validation by means of TEER measurements and FITC-dextran permeability assays. There was an in vivo component of the study which permanently induced ischemic stroke in rats by means of intraluminal middle cerebral artery occlusion (MCAO). To determine if autophagy was increased in OGD cultures, common autophagy markers, LC3-II and p62/SQSTM1, were quantified. Autophagy was not only increased in the OGN cultures, but degradation of the tight junction protein, occludin, was observed. This study showed the direct impact stroke has on the integrity of the BBB and provides in vitro and in vivo data.

To develop innovative treatments for stroke victims, an effective and physiological relevant in vitro model must be created. Due to the recent rise in popularity regarding the use of stem cells in a therapeutic manner, Kim et al.^77^ explored the use of human-derived bone marrow stem cells (hBM-MSCs) and their impact on BBB reconstruction after stroke. The PDMS prepolymer was prepared and then cast into a master mold, created through photolithography. After separating the PDMS piece from the mold, the injection ports and media reservoirs were created using a biopsy punch. The microfluidic chip consisted of five channels, with the center channel, referred to as Channel C, being the main vessel forming channel. What was unique about this design and made it specific to stroke patients was that an angiogenic environment was created using human lung fibroblasts (hLF) as they secrete angiogenic factors such as VEGF. The hLF were seeded in the outer right channel to create an angiogenic concentration gradient. The inner left and right sides were dedicated to media and the difference in media levels determines the spontaneous flow direction throughout the chip. To determine the cellular pattern within the model, the cells were labeled, stained, and then imaged using a fluorescence microscopy. The permeability of the microvessel was determined through time lapsed FTIC images. The main goal of the study was to look at the differential capacity of the hBM-MSCs and how it can impact the pericyte function in post-stroke patients. Western blot analysis exhibited an increase in tight junction proteins, specifically ZO-1 and type IV collagen, in the microvessel model after 7 days post seeding. This was the first study that proved the hBM-MSCs impact the recovery of pericytes within the BBB and provided an efficient in vitro BBB angiogenic chip design.

Thoroughly understanding what exactly causes a pathogenic environment is essential to properly treating CNS disorders, namely stroke. It has been previously proven that there are three neuropeptides, bradykinin (BK), neurotensin (NT), and substrate P (SP), that cause barrier weakening in ischemic stroke patients and there is now evidence that all three peptides have an impact on the permeability of the BBB.^125^ In this study, two separate monolayers were explored, the first being the hCMEC/D3 cell line and then human induced pluripotent stem cell (iPSC)-derived brain microvascular endothelial cells, which were differentiated using conditioned media. Once properly cultured, both cell types were seeded on a TCPS and Transwell inserts which were coated with collagen and fibronectin to promote cell adhesion. A baseline TEER measurement and permeability assay for each insert was taken prior to exposing the monolayers to the neuropeptides in question. Each neuropeptide was dissolved and diluted with EC−/− to obtain the desired concentrations. The first concentration explored for each peptide was 1 µmol/L. For the hDMEC/D3 monolayers, no significant decrease in TEER was observed when exposed to any of the three neuropeptides, but the differentiated iPSC-derived BMECs saw a large decrease in TEER values when exposed to all three types of peptides, but the greatest was seen when exposed to the NT peptide. To observe if the tight junction proteins seen in the monolayers were impacted, protein expression experiments were conducted, specifically looking at claudin-5 and occludin. It was observed that there was no significant decrease in tight junction proteins. Separately, each peptide did not significantly impact the integrity of the BBB, but when combined in varying concentrations, the peptides yielded significant results: there was an increase in fluorescein permeability and decrease in TEER. The final aspect of this study was to observe the effects of neurolysin (Nln), a peptide known to degrade the three pathogenic peptides. Within an in vitro BBB model, Nln was able to degrade and reduce the disease-causing agents of BK, NT, and SP. This was the first study to observe an increase in BBB permeability with direct exposure to NT. Although this study was deemed as a success, there were some shortcomings. First, the use of a monoculture is not the most accurate representation of the BBB, thus further research should be conducted to observe the impact of the neuropeptides when pericytes and astrocytes are also included within the model. Also, the use of Nln as a therapeutic agent should also be further explored, as this could be impactful in the treatment of ischemic stroke.

## Future directions

The in vitro modeling of the BBB not only creates greater understanding of the barrier dynamics, but more recently, the treatment of neurodegenerative diseases. The future work required for BBB modeling will be less focused on model fabrication and validation, and more on how these models can be applied to therapeutic treatments of the brain. Due to the immense need to treat neurodegenerative diseases, the development of brain-permeable delivery options is required. Nanoparticles are a relatively new method in drug delivery as their size, mechanical properties, and material selection are easily altered to the tissue type they are targeted to.^126^ They have come into focus in recent years for drug delivery to the brain due to their noninvasive approach, stability, high drug-loading capacity, and biocompatibility. For drug delivery across the BBB, nanoparticles ranging from polymer based, biomimetic, and inorganic have been explored.^127^ Nanoparticles can be used to load a multitude of cargos, such as anticancer drugs, anti-inflammatory drugs, as well as proteins and genes for gene editing, RNA interference therapy, tissue regeneration, and diagnostics.^128–135^ In addition to nanoparticles, other small molecule therapeutic options are being explored, including gene therapies and the use of liposomes carriers. Finding an optimized treatment option may not happen for many more years due to the wide range of neurological pathologies. However, the treatment options explored in this review provide an impactful start to the research and testing that will be required to continue these advancements. Nanoparticles open the door to endless possibilities in treating brain cancers and neurodegenerative diseases that seemed untreatable in the past due to their tunability and cargo versatility (Figure 7).

Another possibility for future work with BBB is the further use of stem cell-based in vitro models. Several of the reviewed studies show that when properly differentiated, stem cells can be a great source in modeling. It has been seen that stem cells are influenced by their tissue microenvironment,^136^ and that microenvironment can be tuned for many specific differentiation paths through synthetic and natural molecules.^137,138^ The studies that included stem cells in their model often used media that encouraged differentiation and all studies resulted in a successful cell culture. Specifically, work with mesenchymal stem cells (MSCs) is beneficial in BBB studies as, if differentiated properly, promotes angiogenesis, which is essential for BBB modeling.^139^ This also provides the opportunity for patient specific models to be created, allowing for more advanced and individual-based treatments to be developed. The need for patient specific models is seen in studies focused on the treatment of neurodegenerative disease. If a successful patient specific model is paired with a permeable nanoparticle, this is when the most advancement in treatment will be seen. Based on this review, this is where current studies are now focusing, which will be impactful in disease modeling and treatment.

A further step in developing treatment options for the multitude of neurodegenerative diseases and brain cancers is high throughput drug screening. Albeit, many current models fall short due to 2D cultures, which give a poor prognosis for drug-tissue interactions. As mentioned previously, microfluidic devices can alleviate this through their capability for 3D cultures and lead to the development of organ-on-chip or tissue chip models. Microfluidic models have been used to create microvessels, which most closely resemble the blood vessels of the BBB. These are especially attractive because not only are 3D models the most indicative of native tissue behavior, but patient cells from simple biopsies can be cultured for drug screening and personalized treatment plans.^140^ In recent years, tissue chips have become extremely popular in BBB modeling not only for patient specific cultures, but to hopefully eradicate the need for animal studies in drug discovery, especially in industrial settings where high throughput manufacturing, analysis, and implementation are required.^141^ One shortcoming of current studies focusing on microfluidics is that the complexity of the BBB is still not being matched. In addition to microfluidics, newer models, specifically organoids, are being used to mimic the BBB. An organoid is an in vitro 3D miniature representation of a specific organ.^142^ The BBB itself is not modeled as an organoid, but rather, complete brain models are being created and whether or not an in vitro BBB forms as part of the model is being explored.^143^ Just like the previously mentioned models, organoids have some drawbacks, including a lack of complexity due to missing certain cell types, including but not limited to microglial cells.^144^ Future studies must include the fabrication of tricultures exposed to tight junction proteins. In addition, to fully simulate the BBB microenvironment, future models must be dynamic. Cerebral blood flow must be accounted for within a model as this will impact the integrity of the tight junctions. All current in vitro models may not be perfect, but the formulation of an efficient, reproducible, and accurate BBB model that can be adopted by the field as a whole, is necessary for therapeutics to advance exponentially.

## Conclusion

Overall, this review has presented a variety of models that all used different components to accomplish a BBB significant for research. These models not only provide insight on the BBB as a system, but also on a subcellular level. The BBB models explored in this review used a variety of advancements, whether that be moving from a Transwell static model to a microfluidic dynamic model, to the implementation of stem cells in modeling, which may even evolve to the use of patient cells in the future. The advancements made in disease modeling have also created a pathway for the research in drug delivery to the brain. Ultimately, although the BBB is a complex cellular structure, there have been great strides in mimicking it in vitro, which create further opportunities for advancement in brain research.
